# Tetracyclic 1,4-Naphthoquinone Thioglucoside Conjugate U-556 Blocks the Purinergic P2X7 Receptor in Macrophages and Exhibits Anti-Inflammatory Activity In Vivo

**DOI:** 10.3390/ijms241512370

**Published:** 2023-08-02

**Authors:** Sergei Kozlovskiy, Evgeny Pislyagin, Ekaterina Menchinskaya, Ekaterina Chingizova, Leonid Kaluzhskiy, Alexis S. Ivanov, Galina Likhatskaya, Irina Agafonova, Yuri Sabutski, Sergey Polonik, Igor Manzhulo, Dmitry Aminin

**Affiliations:** 1 G.B. Elyakov Pacific Institute of Bioorganic Chemistry, Far Eastern Branch of the Russian Academy of Science, 690022 Vladivostok, Russia; sergeimerx@gmail.com (S.K.); pislyagin@hotmail.com (E.P.); ekaterinamenchinskaya@gmail.com (E.M.); martyyas@mail.ru (E.C.); galin56@mail.ru (G.L.); agafonova@piboc.dvo.ru (I.A.); alixar2006@yandex.ru (Y.S.); sergpol@piboc.dvo.ru (S.P.); 2 V.N. Orekhovich Institute of Biomedical Chemistry, 119121 Moscow, Russia; leonid.kaluzhskiy@ibmc.msk.ru (L.K.); professor-ivanov@yandex.ru (A.S.I.); 3 A.V. Zhirmunsky National Scientific Center of Marine Biology, Far Eastern Branch of the Russian Academy of Science, 690041 Vladivostok, Russia; i-manzhulo@bk.ru

**Keywords:** 1,4-naphthoquinone, P2X7 receptor, Ca^2+^ influx, YO-PRO-1 uptake, ROS production, macrophages, inflammation, SPR analysis, molecular docking

## Abstract

P2X7 receptors (P2X7Rs) are ligand-gated ion channels that play a significant role in inflammation and are considered a potential therapeutic target for some inflammatory diseases. We have previously shown that a number of synthetic 1,4-naphthoquinones are capable of blocking P2X7Rs in neuronal and macrophage cells. In the present investigation, we have demonstrated the ability of the tetracyclic quinone-thioglucoside conjugate **U-556**, derived from 1,4-naphthoquinone thioglucoside, to inhibit ATP-induced Ca^2+^ influx and YO-PRO-1 dye uptake, which indicates blocking P2X7R in RAW 264.7 macrophages. This process was accompanied by the inhibition of ATP-induced reactive oxygen species production in macrophages, as well as the macrophage survival strengthening under ATP toxic effects. Nevertheless, **U-556** had no noticeable antioxidant capacity. Naphthoquinone-thioglucoside conjugate **U-556** binding to the extracellular part of the P2X7R was confirmed by SPR analysis, and the kinetic characteristics of this complex formation were established. Computer modeling predicted that **U-556** binds the P2X7R allosteric binding site, topographically similar to that of the specific A438079 blocker. The study of biological activity in in vivo experiments shows that tetracylic conjugate significantly reduces inflammation provoked by carrageenan. The data obtained points out that the observed physiological effects of **U-556** may be due to its ability to block the functioning of the P2X7R.

## 1. Introduction

The purinergic P2X7 receptor (P2X7R) is a trimeric ligand-gated cation channel that is activated by extracellular adenosine triphosphate (ATP). Receptor activation is accompanied by a rapid Na^+^ and Ca^2+^ influx into and K^+^ efflux out of the cell cytoplasm, as well as the opening of large pores (macropores) to facilitate the passage of organic ions and hydrophilic solutes of MW up to 900 Da. This property of P2X7R distinguishes them from other subtypes of the P2X receptor family and allows the use of a number of fluorescent molecules, including ethidium bromide, YO-PRO-1, or Lucifer yellow, to assess the functioning of the macropore [1,2]. The opening of the P2X7R macropore in response to the ATP action triggers some cellular processes, such as the release of cathepsins, the production of reactive oxygen species (ROS), the plasma membrane blebbing, and apoptosis, while prolonged activation of the receptor leads to cell death [3].

This type of receptor is expressed by different types of cells, including dendritic and mast cells, T and B lymphocytes and their precursors, macrophages, and microglia. Because the P2X7R is highly expressed in immune cells, its signaling is important for modulating both the innate and adaptive immune response and, in particular, for the regulation of inflammation [3,4]. Dysfunction or overexpression of P2X7R causes numerous pathological conditions associated with pain and inflammation. It is known that P2X7R activates the assembly of the NLRP3 inflammasome, control the release of pro-inflammatory cytokines and the activation of immune cells, thereby modifying immune and inflammatory responses, which leads to a wide range of diseases [4].

Currently, the search and development of P2X7R blockers with high selectivity, efficacy, and bioavailability is an urgent pharmacological task. A series of P2X7R blockers with high inhibitory activity has already been synthesized. Several P2X7R antagonist compounds are in various stages of preclinical and clinical trials. However, despite the intensive search for highly selective P2X7R blockers, as well as modulators of other receptors of this family, so far, none of them has been brought to the pharmaceutical market [1,5].

1,4-Naphthoquinones (1,4-NQs) are a class of natural and synthetic compounds with a broad variety of biological activities, including analgesic and anti-inflammatory properties [6]. It has been proven that some synthetic derivatives of the 1,4-naphthoquinoid nucleus are able to effectively bind P2X7 receptor and inhibit its functions in in vitro, in vivo, and in silico models [7,8,9,10,11]. As a result, this class of compounds has been considered the scaffold for the development of effective drugs blocking the functions of P2X7R.

This work continues a series of studies of the biological activity of a number of 1,4-naphthoquinones synthesized by us and their ability to influence the functioning of purinergic P2X7 receptors in various cell types. We have already evaluated the effect of these compounds on Neuro-2a neuronal cells [12] and the effect of some of them on the functioning of P2X7R in macrophage cells [13]. In this study, we focused on the extended investigation of the synthetic tetracyclic thioglucoside conjugate, **U-556**, impact on RAW 264.7 macrophage P2X7R functioning, data for which have not been presented before. Herein, we report that this compound is able to inhibit ATP-induced Ca^2+^ influx and YO-PRO-1 uptake into mouse macrophage cells, reduce ROS production, and protect macrophages from the cytotoxic ATP impact. Above all, the novelty of this work is new data on the effect of **U-556** on the functioning of the receptor macropore upon modulation by a specific P2X7R agonist and antagonist. The work expands studies of the cytoprotective effect of naphthoquinone in the toxic effect of high concentrations of ATP. Direct binding of **U-556** to P2X7R was confirmed by surface plasmon resonance (SPR). Finally, the anti-inflammatory activity of **U-556** in in vivo experiments was carried out in a model of carrageenan-induced paw edema in mice.

## 2. Results

### 2.1. Synthesis of the Tetracyclic Naphthoquinone Thioglucoside Conjugate U-556

**Figure 1:**
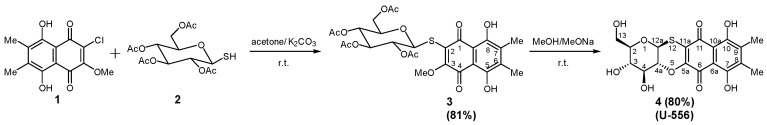
Scheme of the synthesis of tetracyclic quinone-thioglucoside conjugate **U-556**.

Synthesis of tetracyclic quinone-thioglucoside conjugate **U-556** was developed according to our works [12,14]. Briefly, 2-chloro-5,8-dihydroxy-3-methoxy-6,7-dimethyl-1,4-naphthoquinone **1** was condensed with tetra-O-acetyl-1-thio-β-D-glucose **2**, and acetylthioglucoside of 1,4-naphthoquinone **3** was converted to the tetracyclic quinone-thioglucoside conjugate **4 U-556** by treatment with MeONa. Deacetylation of sugar moiety in the thioglucoside **3** is accompanied by intramolecular substitution of the MeO- group in the quinoid nucleus and the formation of the tetracyclic quinone-carbohydrate conjugate **4**. The total scheme of synthesis is shown in Figure 1.

### 2.2. U-556 Inhibits ATP-Induced Ca^2+^ Influx in Macrophage Cells

**Figure 2:**
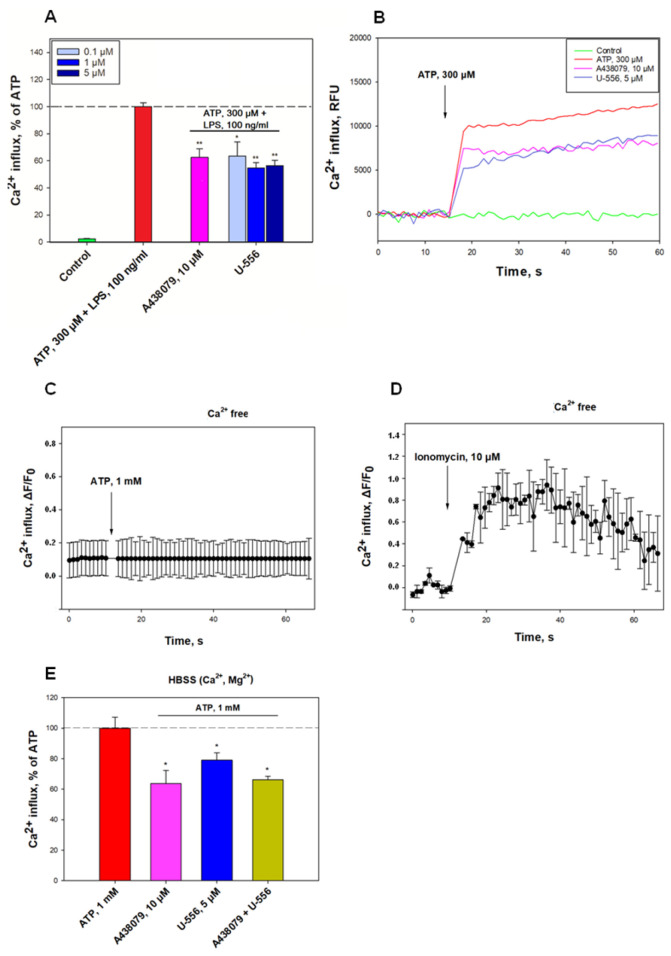
Quinone-thioglucoside conjugate **U-556** inhibits ATP-induced Ca^2+^ influx in RAW 264.7 cells. (**A**) Effect of **U-556** (0.1, 1.0, and 5.0 µM) and A438079 (10 µM) on Ca^2+^ influx induced by ATP (300 μM) in cells primed with LPS (100 ng/mL). Data are presented as mean ± SEM (n = 3); * *p* < 0.05, ** *p* < 0.01 compared with ATP+LPS. (**B**) Representative curves of [Ca^2+^]_i_ elevation induced by ATP (300 μM) and LPS (100 ng/mL) alone and in the presence of **U-556** or A438079. (**C**,**D)** Representative traces of [Ca^2+^]_i_ increase induced by ATP 1 mM alone and ionomycin (10 μM) introduced after ATP 1 mM application in Ca-free medium. (**E**) Calculated increase in [Ca^2+^]_i_ in macrophages caused by ATP 1 mM alone, in the presence of **U-556** 5 μM or A438079 10 μM, or in a combination of **U-556** with A438079. The data are presented as the mean ± SEM (n = 6); * *p* < 0.05 compared with ATP.

The first objectives of the study were to establish the ability of 1,4-NQ derivative **U-556** to inhibit the functions of the P2X7R in RAW 264.7 macrophages. For this purpose, cells were loaded with a Ca^2+^-sensitive Fluo-8AM fluorescent probe. After the addition of ATP to macrophages primed with LPS, a significant elevation of intracellular calcium concentration ([Ca^2+^]_i_) was registered. The selective P2X7R blocker A438079 noticeably inhibited calcium influx by 37.5 ± 6.4%, respectively (Figure 2).

Treatment of cells with **U-556** at concentrations of 0.1, 1.0, and 5.0 μM resulted in dose-dependent decline of calcium entry by 36.7 ± 10.6%, 45.4 ± 4.1%, and 43.6 ± 4.0%, correspondingly. The data obtained indicate a direct inhibitory effect of the studied compound on the P2X7R ion channel functioning comparable to that of an A438079 blocker (Figure 2).

In additional experiments, the cells were cultured in a Ca^2+^-free medium. In these conditions, ATP did not elevate [Ca^2+^]_i_ (Figure 2C), but the application of the calcium ionophore ionomycin followed by ATP led to a perceptible rise in intracellular Ca^2+^ concentration (Figure 2D). Besides, the mutual use of **U-556** with A438079 did not lead to a decrease in the Ca-responses of cells to high concentrations of ATP in comparison with either of these compounds in a complete medium (Figure 2E). This indicates the absence of an additive effect for 1,4-NQ derivative and the P2X7R blocker.

### 2.3. U-556 Inhibits ATP-Induced YO-PRO-1 Dye Entry in Macrophage Cells

**Figure 3:**
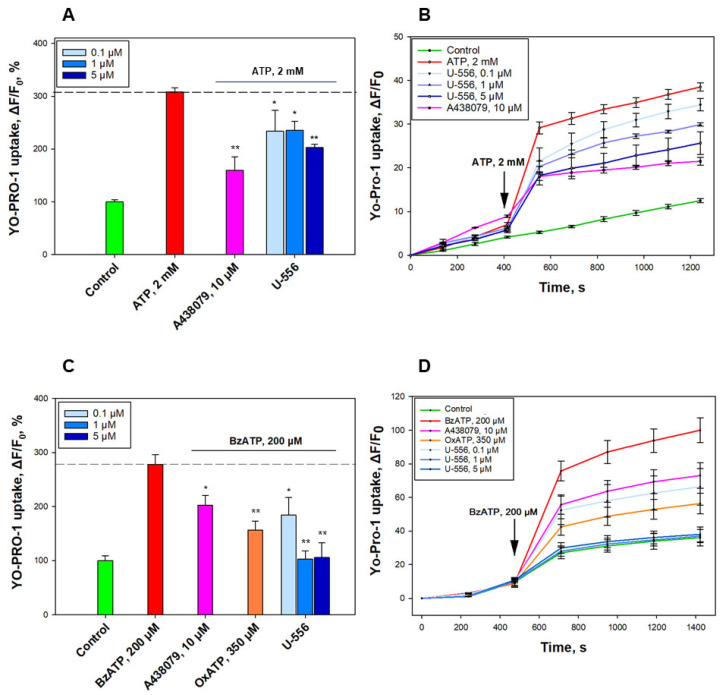
Quinone-thioglucoside conjugate **U-556** inhibits ATP- and BzATP-induced uptake of YO-PRO-1 in RAW 264.7 cells. Effect of **U-556** (0.1, 1.0, and 5.0 µM), A438079 (10 µM), and OxATP (350 μM) on YO-PRO-1 uptake induced by 2 mM ATP or 200 μM BzATP (**A**,**C**). Representative curves showing the uptake of YO-PRO-1 dye in the presence of **U-556,** A438079, and OxATP induced by ATP or BzATP (**B**,**D**). Data are presented as mean ± SEM (n = 6); * *p* < 0.05, ** *p* < 0.01 compared to ATP or BzATP.

To assess the ability of **U-556** to block the formation of P2X7R macropores in macrophage cells, a low molecular weight fluorescent dye YO-PRO-1 was used. Under ATP influence, a conspicuous increase in the dye uptake into the macrophages was found, amounting to 208.3 ± 7.9%, relative to the control without ATP. The effect of the investigated 1,4-NQ derivative on dye uptake was studied in comparison with the A438079 blocker, which significantly reduced dye entry by 48.3 ± 8.3% (Figure 3A,B). Incubation of macrophage cells with compound **U-556** at concentrations of 0.1, 1.0, and 5.0 μM resulted in a partial inhibition of dye uptake by 24.3 ± 12.9%, 23.7 ± 5.6%, and 34.3 ± 2.1%, accordingly (Figure 3A,B).

To confirm the inhibitory effect of **U-556** on the functioning of the P2X7 receptor macropore, we used a selective P2X7 purinergic receptor agonist, 2,3-O-(4-benzoylbenzoyl)ATP (BzATP). This agonist sharply increased the entry of YO-PRO-1 into cells by around three-fold compared to the control cells. A438079 blocker and P2X receptors antagonist, oxidized ATP (OxATP), significantly reduced dye uptake by 42.2 ± 10.3% and 68.2 ± 9.4%, respectively (Figure 3C,D). At the same time, **U-556** at a concentration of 0.1 μM eliminated YO-PRO-1 uptake by 52.7 ± 18.4%, and at concentrations of 1.0 and 5.0 μM compound significantly suppressed dye uptake almost to the baseline level of control cells by 98.6 ± 8.6% and 97 ± 15.3% (Figure 3C,D).

### 2.4. U-556 Inhibits ATP-Induced ROS Production in Macrophage Cells

**Figure 4:**
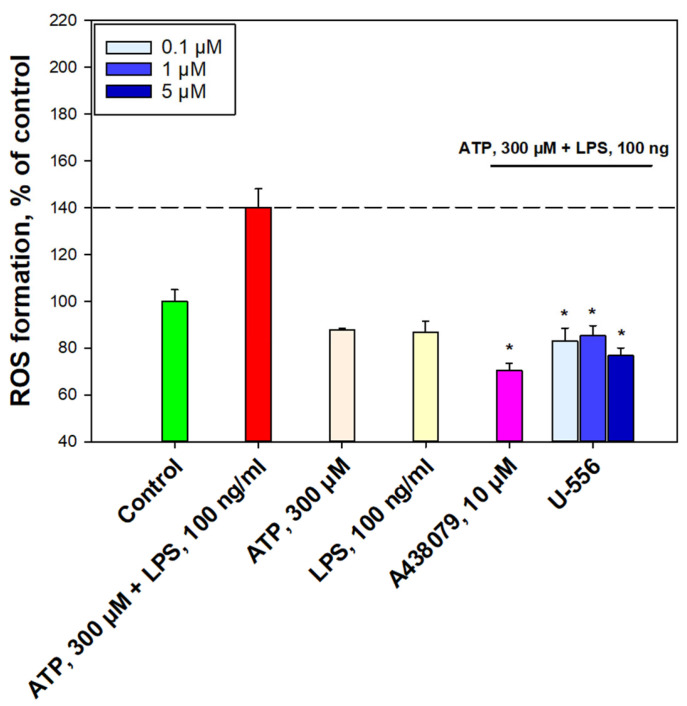
Quinone-thioglucoside conjugate **U-556** reduces ATP-induced ROS production in RAW 264.7 cells. Data are presented as mean ± SEM (n = 3); * *p* < 0.05 compared to ATP+LPS.

To examine further the role of **U-556** in P2X7R functioning, the ability of this 1,4-NQ derivative to decrease ATP-induced production of ROS in RAW 264.7 macrophages primed with LPS was evaluated. Macrophage priming led to an elevation of ROS amount in cells in response to the ATP action by 40.2 ± 8.0%. At the same time, cell treatment with either ATP or LPS alone did not show a significant magnification in intracellular ROS level. This may indicate a synergistic involvement of these two compounds in the induction of oxidative stress and inflammation (Figure 4).

The inhibitory effect of the studied compound was evaluated in comparison with A438079, which at a concentration of 10 µM, suppressed ROS formation by 69.7 ± 3.2%. Pretreatment of cells with compound **U-556** at concentrations of 0.1, 1.0, and 5.0 μM resulted in a pronounced reduction of intracellular ROS amount by 57.2 ± 5.6%, 54.9 ± 4.3%, and 63.3 ± 3.2%, respectively (Figure 4).

### 2.5. U-556 Protects Macrophage Cells from ATP Toxicity

To argue additionally that the **U-556** interacts with P2X7R and assess how this interaction is related with ATP cytotoxic action on macrophage survivability, we used four different approaches. The level of lactate dehydrogenase release from dead cells was determined (LDH test), the activity of intracellular enzyme nonspecific esterase using a fluorescein diacetate probe was detected (FDA test), the MTT assay was used to assess the influence on cell metabolism and biomembrane integrity using dye exclusion assay was evaluated (Trypan blue test).

**Figure 5:**
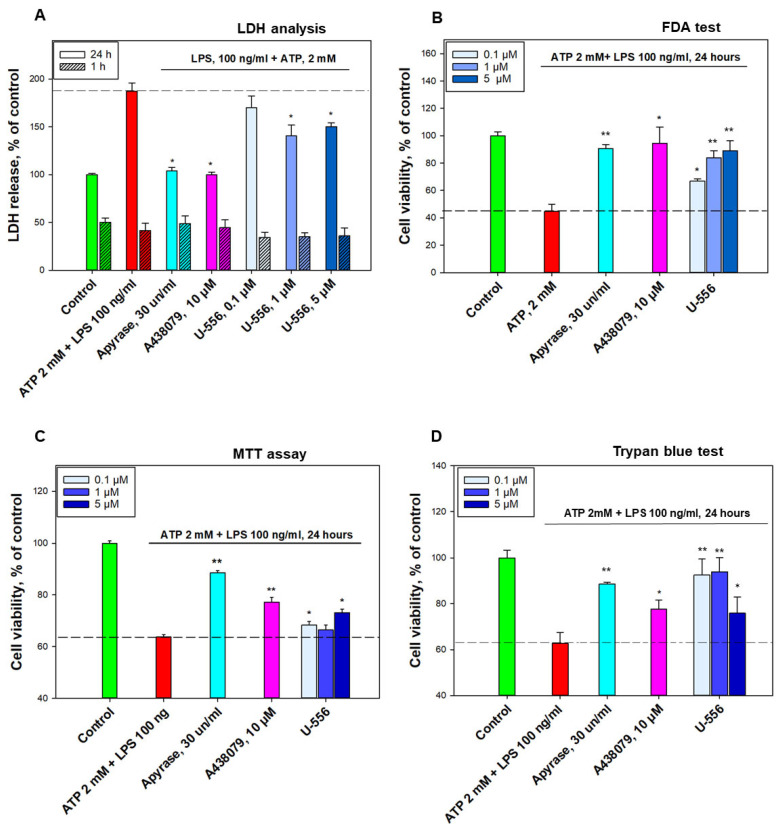
Quinone-thioglucoside conjugate **U-556** protects RAW 264.7 cells from the toxic effects of ATP/LPS. The effect of 1,4-NQ derivative on cell viability under the action of ATP (2 mM)/LPS (100 ng/mL) was assessed using: (**A**) LDH analysis, (**B**) FDA test, (**C**) MTT assay, (**D**) Trypan blue test. Data are presented as mean ± SEM (n = 6 for (**A**,**B**); n = 3 for (**C**,**D**)); * *p* < 0.05, ** *p* < 0.01 compared to ATP+LPS.

The cytotoxic effect of ATP/LPS upon macrophages after 24 h of incubation was expressed primarily in a noticeable increment of LDH level in the extracellular medium by 87.1 ± 8.6% relative to the control, as well as the nonspecific esterase inhibition activity by 50.2 ± 6.5%, detected in the FDA test (Figure 5A,B). This process was accompanied by a decrease in the number of viable cells, which was detected by the MTT assay and Trypan blue exclusion assay, the percentage of which was diminished on 36.3 ± 1.0% and 37.3 ± 4.8%, correspondingly (Figure 5C,D).

To reveal the role of P2X7R in cell death, in this experiment, we used the enzyme apyrase, which completely removed ATP from the extracellular medium due to its hydrolysis, and the specific A438079 blocker. It was found that under these conditions, in the presence of ATP, the viability of cells treated with apyrase or A438079 corresponded to or was very close to the cell viability level of the intact control (Figure 5A–D).

Compound **U-556** has a low cytotoxicity [12]. Using the LDH analysis, it was shown that this 1,4-NQ derivative promotes a significant suppression of LDH release from macrophages in the presence of ATP/LPS after 24 h of incubation. At concentrations of 0.1, 1.0, and 5.0 μM, this conjugate showed an effective decrease in the protease content in the extracellular medium by 17.3 ± 12.4%, 46.4 ± 11.1%, and 37.1 ± 4.1%, correspondingly (Figure 5A). At the same time, we did not find any significant changes in the LDH release from cells 1 h after ATP/LPS administration. Under these conditions, no protective effects of **U-556** and A438079 were observed. Therefore, in further studies of cytoprotection, we used only 24 h to incubate cells with effectors. Analysis of cell viability in the FDA test produced results similar to LDH analysis results. Thus, **U-556** at concentrations of 0.1, 1.0, and 5.0 μM augmented the viable cell number in the presence of ATP/LPS by 22.3 ± 1.5%, 39.1 ± 5.3%, and 44.3 ± 7.4%, accordingly (Figure 5B). Thus, compound **U-556** perceptibly protected macrophages pre-primed with LPS in the presence of a cytotoxic concentration of ATP. Using the MTT assay, it was shown that this 1,4-NQ promotes macrophage survival in the presence of cytotoxic amount of ATP/LPS. Studied **U-556** at concentrations of 0.1, 1.0, and 5.0 µM fractionally increased cell metabolic activity on 7.3 ± 2.3%, 4.3 ± 3.0%, and 14.9 ± 2.2%, respectively (Figure 5C). During the Trypan blue exclusion assay, it was found that **U-556** substantially preserves the biomembrane integrity and, accordingly, cell viability, in the presence of ATP/LPS. At concentrations of 0.1, 1.0, and 5.0 µM, this 1,4-NQ retained cell viability at the level of 92.6 ± 7%, 94.0 ± 6.3%, and 75.9 ± 7.1%, respectively (Figure 5D).

### 2.6. U-556 Does Not Exhibit Antioxidant Activity

**Figure 6:**
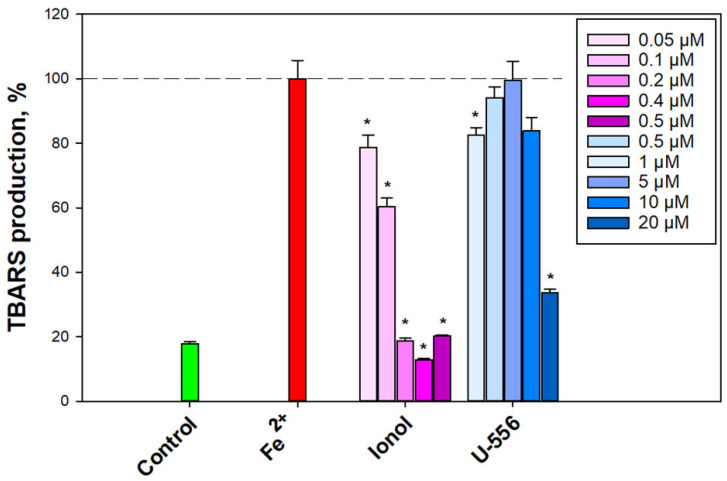
Effect of quinone-thioglucoside conjugate **U-556** on Fe^2+^-induced oxidation of mouse brain homogenate. Mouse brain homogenate oxidation was induced using FeSO_4_, and the content of TBARS was determined in the presence of ionol (positive control) and 1,4-NQ derivative by spectrofluorimetry. Data are presented as mean ± SEM (n = 3); * *p* < 0.05 compared to Fe^2+^.

Taking into consideration that some 1,4-NQs are known to have noticeable antioxidant properties [6], we inspected the antioxidant activity of compound **U-556** in a model of non-enzymatic Fe^2+^-induced oxidation of a mouse brain homogenate. The introduction of a peroxidation inducer into the brain homogenate led to a substantial magnification of the final oxidation products that react with thiobarbituric acid (TBARS) (Figure 6).

Pre-incubation of the brain homogenate with compound **U-556** in the concentration range of 0.5–10.0 µM resulted in an inconspicuous effect and very weak elimination in the TBARS level. The antioxidant capacity of the studied quinone-thioglucoside conjugate **U-556** at the highest used concentration of 20.0 μM was significantly lower than that of the control antioxidant ionol at a concentration of 0.2 μM (Figure 6).

Moreover, the investigated quinone-thioglucoside conjugate **U-556** did not show any considerable diphenylpicrylhydrazyl (DPPH) free radical scavenging. The maximum binding of free radicals around 48% was shown at a maximal studied concentration of 100.0 μM (Table 1), while the standard antioxidants, quercetin, and ascorbic acid, used as reference compounds, showed pronounced antioxidant properties with an EC_50_ of 41.75 ± 1.71 µM and 9.38 ± 0.42 µM, respectively.

### 2.7. U-556 Binds to P2X7R

In order to establish the direct interaction of the studied **U-556** with the P2X7R, the SPR approach was utilized. For this purpose, the extracellular domain of the recombinant P2X7 receptor (36.8 kDa) was immobilized on the chip of the Biacore 3000 optical biosensor (with a density of 12.3 ng of protein/mm^2^), and the target quinone-thioglucoside conjugate **U-556** was passed in a stream of the HBS-N buffer solution to bind the analyte to ligand.

**Figure 7:**
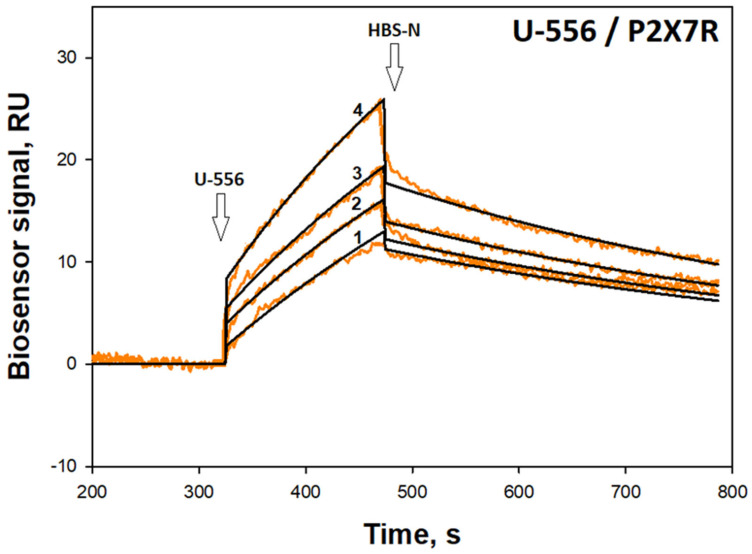
Sensorgrams of surface plasmon resonance binding obtained for immobilized P2X7R after **U-556** application at 25 °C by 2.5 μM concentration (curve 1), 5.2 μM (curve 2), 6.1 μM (curve 3), and 8.4 μM (curve 4). Fitting curves (theoretical model) drawn in black. The time of 1,4-NQ and HBS-N buffer injection is indicated by arrow.

Dose-response curves were obtained (Figure 7) and the kinetic characteristics of the U-556/hP2X7R complex were estimated. The dissociation constant (Kd) of the complex was calculated as 13.0 μM. All kinetic parameters of the complex are presented in Table 2.

### 2.8. Molecular Docking

A full atom model of the mouse P2X7 receptor homotrimer was obtained, as described in [12]. The 3D structure of the mP2X7R was simulated, and a model of the mR2X7R trimer was created based on the rat P2X7R structure as a template (PDB ID 6U9V). The molecular complexes between the studied compounds, including all tautomeric forms and the mP2X7R model, were predicted using the structural bioinformatics and molecular docking approach.

**Figure 8:**
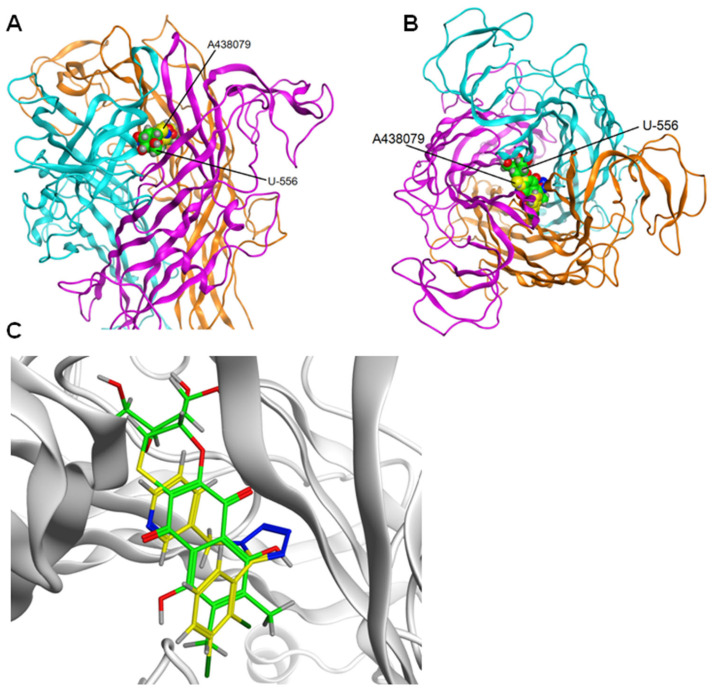
Extracellular domain of the mouse P2X7 receptor and compounds **U-556** and A438079 at the allosteric site of the receptor. The structure of the receptor is shown as ribbon, compounds **U-556** and A438079 are shown as spacefill in green and yellow, respectively. (**A**) Side view of the receptor and (**B**) top view of the receptor. (**C**) Superposition of binding sites for compounds **U-556** and A438079 in the allosteric site of the receptor. Compounds **U-556** and A438079 are shown as sticks in green and yellow, respectively.

The docking results for the **U-556** and the extracellular domain of mP2X7R showed that the studied quinone-thioglucoside conjugate has an energetically favorable binding pose in the allosteric site. Then, contact analysis of 5 complexes of **U-556** with mP2X7R was carried out for the most energy-efficient complexes of **U-556** in the allosteric site. It was found that the studied compound is localized inside the allosteric site pocket located between the subunits of the extracellular domain (Figure 8A,B). For all calculated complexes, the binding energy was negative.

There are 3 allosteric binding sites in the ectodomain of P2X7R formed between two adjacent subunits in a trimer. The sites have the same structure. One of the sites between subunits B and C of P2X7R was arbitrarily chosen to calculate the complexes. The docking results for the **U-556** and the extracellular domain of mP2X7R showed that the studied quinone-thioglucoside conjugate has an energetically favorable binding pose in the allosteric site with binding energy −7.4 kcal/mol.

Analysis of the docking results showed that the superposition of the binding sites of the studied compounds **U-556** with mP2X7R and the P2X7R-specific allosteric site of the A438079 inhibitor pointed that these sites partially overlapped (Figure 8C). Nevertheless, as shown in Table 3, quinone-thioglucoside conjugate **U-556** binds energetically more preferably to the P2X7R allosteric site (E = −5.6 kcal/mol) than the selective antagonist A438079 (E = −2.6 kcal/mol). Compound **U-556** forms H-donor and H-acceptor contacts with Ala296_C and H-acceptor contact with Ala296_B, while A438079 interacts only with Ala296_B.

**Figure 9:**
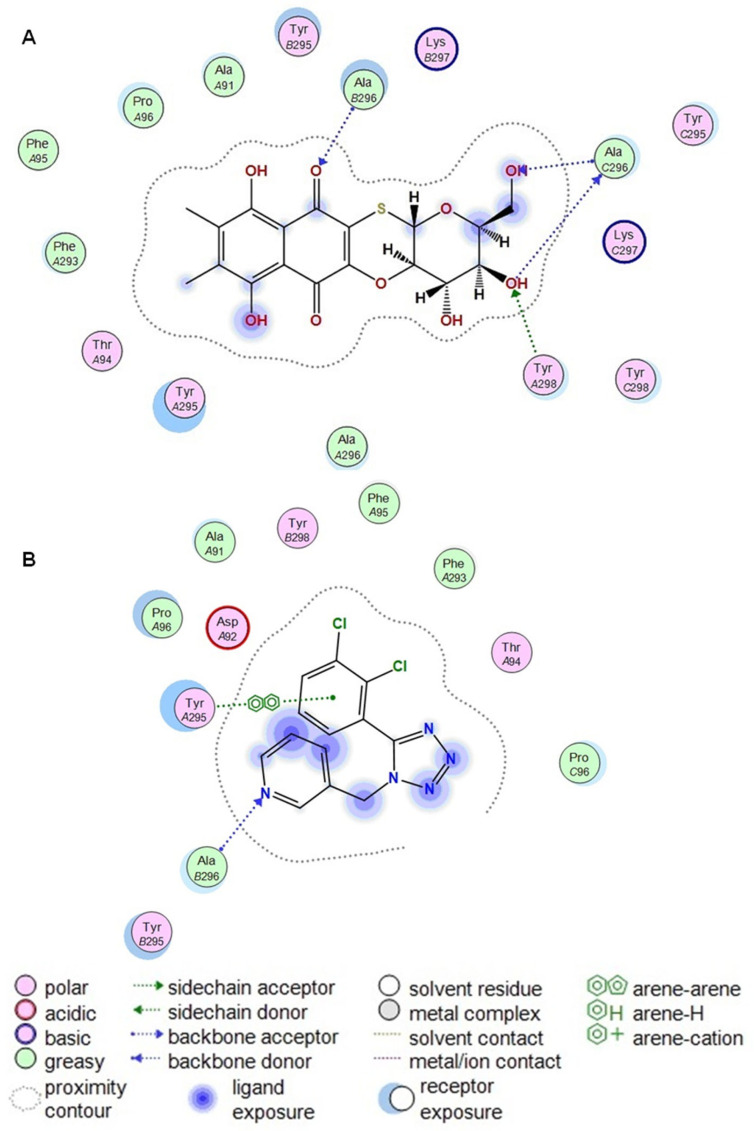
2D diagrams of contacts between the complexes of the murine P2X7 receptor compound **U-556** (**A**) and the selective antagonist A438079 (**B**).

Figure 9 shows the most energetically beneficial possible poses of **U-556** and A438079 in a complex with mP2X7R. The strongest binding of both compounds, **U-556** and A438079, is observed with the amino acid residue Ala296 of subunit B (Figure 9 and Table 3).

### 2.9. U-556 Inhibits Paw Edema

**Figure 10:**
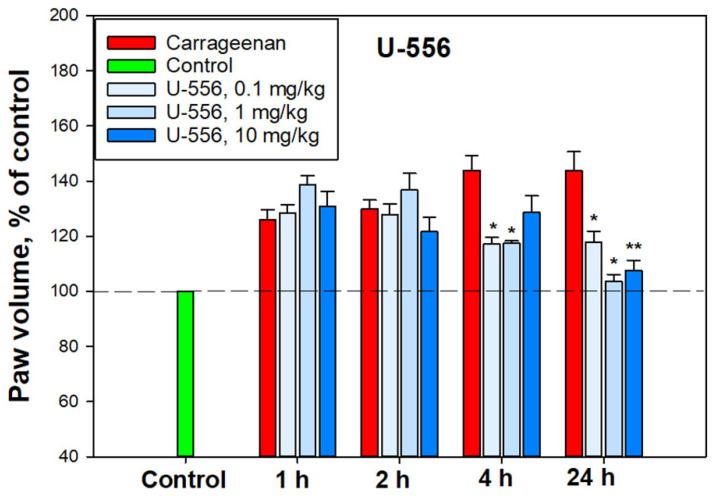
Anti-inflammatory effect of quinone-thioglucoside conjugate **U-556** in a model of carrageenan-induced paws edema. A solution of conjugate **U-556** was administered intraperitoneally 60 min before the induction of inflammation. Paw volume was measured 1, 2, 4, and 24 h after carrageenan intraplantar injection at a concentration of 1.5 mg/mL in the volume of 20 μL. Results are presented as mean ± SEM (n = 6); * *p* < 0.05, ** *p* < 0.01 compared to carrageenan.

The anti-inflammatory activity of compound **U-556** was studied in an in vivo mouse paw edema model. A single intraplantar administration of carrageenan caused conspicuous edema, expressed as enlarged mouse paw volume. The greatest edema was observed 4 and 24 h after the injection of the pro-inflammatory agent (Figure 10).

Compound **U-556** had a weak anti-inflammatory effect in the first 1–2 h, but after 4 h, a pronounced, significant, and dose-dependent lessening in paw edema was observed. At doses of 0.1, 1.0, and 10.0 mg/kg, this quinone-thioglucoside conjugate **U-556** reduced inflamed paw volume by 60.8 ± 5.4%, 60.3 ± 2%, and 34.8 ± 13.6%, respectively, relative to animals injected with carrageenan only. Twenty-four hours post **U-556** application at doses of 1.0 and 10.0 mg/kg, the edema was almost completely neutralized up to the control values of paw volume in intact animals. In these conditions, the mouse paw inflammation was significantly minimized by 91.7 ± 5.6% and 82.7 ± 8.4% (Figure 10).

## 3. Discussion

Macrophages (or the “big eaters”) are specialized cells in the body of animals and humans capable of actively capturing and digesting bacteria, the remains of dead cells, and other particles that are foreign or toxic to the body. Macrophages are present in virtually every organ/tissue, where they act as the first line of immune defense against pathogens and play an important role in maintaining tissue homeostasis, and are directly involved in the processes of inflammation [15]. One of the key targets of the signaling pathway leading to inflammation is the P2X7 receptor of macrophages. Activation of P2X7R by high concentrations of ATP under pathological conditions provides a non-selective flow of a number of mono- and divalent cations and some low-molecular chemicals through the formed receptor macropore. This process is coupled in macrophages with LPS-recognizing toll-like receptor 4 (TLR4), which co-activation leads to the formation of inflammasome 3 (NLRP3), followed by activation of (pro-IL-1β)-degrading caspase-1, and release of the inflammatory inducer, the pro-inflammatory cytokine IL-1β [16].

It has now become clear that a number of inflammatory diseases, such as rheumatoid arthritis, Crohn’s disease, liver fibrosis, sepsis, kidney and lungs inflammation, tumor-associated inflammation, and some other, is largely due to the activation of P2X7R in macrophages. The same pattern is found in the study of the causes of some neurodegenerative disorders related to neuroinflammation, such as Alzheimer’s disease, Parkinson’s disease, Huntington’s disease, amyotrophic lateral sclerosis, multiple sclerosis, postischemic conditions, and epilepsy. The important role of P2X7R in the processes of inflammation has led to an intensive search for highly effective selective blockers of this target. However, despite the fact that a number of pharmaceutical companies have already created a series of promising ligands with pronounced P2X7R inhibitory properties, none of these compounds has successfully passed clinical trials yet [4,17].

The anti-inflammatory properties of 1,4-NQs are well known. Some of the natural and synthetic compounds were found to inhibit histamine, IL-1β, IL-6, and TNF-α release, cyclooxygenase-2 (COX-2) activity, and reduce edema in the carrageenan model of inflammation [6]. Some synthetic 1,4-NQs were observed to effectively block the P2X7R functions in mouse neuronal and macrophage cells [7,8,9,10,11,12,13]. In this study, we proved that synthetic tetracyclic naphthoquinone-thioglucoside conjugate **U-556**, is able to effectively block the functioning of P2X7R in mouse macrophages. This effect manifested itself primarily in the inhibition of ATP-induced Ca^2+^ influx into macrophage cells primed with bacterial LPS. The response of macrophages to LPS is associated with the triggering of a cascade of pro-inflammatory reactions, mainly through the TLR4 receptor which stimulates additional ATP release and enhances the P2X7R response [18]. Moreover, such stimulation, in turn, increases the percentage of death cells exposed to ATP. The level of ion transport inhibition for **U-556** was comparable to the selective P2X7R blocker A438079.

To exclude the participation of P2Y receptors also present in macrophages, the ATP-induced changes in intracellular calcium concentration Ca^2+^ transport was measured in a calcium-free medium. Under these conditions, ATP did not cause an increase in [Ca^2+^]_i_. Since metabotropic P2Y receptors are not able to provide calcium current from the extracellular medium through cell membranes, unlike P2X, the participation of P2Y receptors was excluded. Ionomycin is a selective calcium ionophore that transports Ca^2+^ through biomembranes along a concentration gradient. In a calcium-free medium, ionomycin increases [Ca^2+^]_i_ in the cytoplasm due to Ca^2+^ transport from intracellular depots. In our study, the use of the Ca-selective ionophore ionomycin in Ca^2+^-free media resulted in a marked elevation of [Ca^2+^]_i_. This suggests that intracellular calcium stores remain full after ATP application and are not involved in the Ca-response of cells to the ATP.

A significant increase in the YO-PRO-1 uptake into macrophages in the presence of ATP confirms the opening of the P2X7R macropore under these conditions. The data obtained on reducing the dye uptake after pre-incubation of cells with **U-556** corroborate a direct inhibitory effect of the studied conjugate **U-556** on the ATP-induced P2X7R macropore formation compatible with A438079 blocker action. Experiments with a selective P2X7R agonist convincingly demonstrate a pronounced entry of the dye through the macropores of this receptor formed under BzATP action. Noteworthy, under these conditions, **U-556** actually completely suppresses the dye uptake by cells, indicating a complete blocking of the P2X7R function. Surprisingly, the selective blocker A438079 and P2X receptor antagonist OxATP did not show a complete blocking effect on the functioning of the receptor macropore. This is probably due to the peculiarities of the structure or functioning of P2X7R in RAW 264.7 macrophage cells.

The ability of the investigated conjugate **U-556** to significantly suppress the elevation of ROS production in cells and to protect and maintain the macrophage metabolic activity and viability in the presence of a cytotoxic ATP concentration proves that this compound can block the formation of P2X7R macropores the opening of which leads to cell death due to oxidative stress. However, no significant anti-radical activity of the studied compound was observed in the DPPH test and in the test of peroxidation in the mouse brain homogenate. This suggests that the studied conjugate **U-556** has no pronounced antioxidant power and does not additionally contribute to diminishing intracellular ROS levels under ATP influence. A diminution in the ATP cytotoxic effect expressed as the elimination of the biomembrane integrity, permeability disturbance, and LDH release from cells, as well as in the abolition of the intracellular esterase inhibition, an increase in cell metabolic activity is an additional verification of compound **U-556** capability to retain the viability of macrophages by blocking of P2X7R functioning.

In this study, we did not find a pronounced dose-effect relationship for compound **U-556**. The same lack of clear dose dependence has been previously described for other 1,4-NQs, which is likely due to the non-linear mode of P2X7R inhibition [12,13].

Compound **U-556** selective binding to P2X7 subunits and the formation of a stable complex was demonstrated by SPR. Indeed, this is direct evidence of the ability of the studied 1,4-NQ derivative to specifically interact with the extracellular part of the P2X7 subtype purine receptors, which can affect their functioning. The parameters of naphthoquinone/P2X7R interaction estimated using the SPR technique may differ from those observed in experiments with live cultured macrophages since the conditions for these two types of studies are significantly different.

We showed that quinone-thioglucoside conjugate **U-556** can penetrate the allosteric binding site and interact with the inner part of the pocket. The contacts between the quinone-thioglucoside conjugate core and Ala296_C and Ala296_B amino acid residues seem to be the most substantial contributors to this reciprocity. Computer modeling predicted that **U-556** binds the P2X7R allosteric binding site, topographically similar to that of the specific A438079 blocker. The failure in an additive effect of the inhibitory action of **U-556** and A438079 when used in combination (Figure 2E) is the further verification of a single allosteric binding site existence for these two compounds to the P2X7 receptor.

At present, the crystal structure of the complex of A438079 with P2X7R has not been established. Work [19] showed that antagonists with different structures bind to the same P2X7R allosteric site. Depending on the structure of antagonists, various contacts are possible at the allosteric site. At the same time, there are papers in which A438079 was docked to the allo- and orthosteric P2X7R sites [20]. The allosteric site has been shown to be the most beneficial.

Amino acid residues Phe88, Ile310, Met105, Phe103, Phe293, Tyr295, Tyr298, Asp92, Glu73, Lys110, and Lys297 are the residues that form the allosteric site of the P2X7 receptor. The set of these residues is the same both in article [12] and in this study, there are no differences. However, not all these residues form H-donor and H-acceptor contacts with **U-556**. Previously, we noted that **U-556** forms contacts with Asp92 and Glu73. At the same time, in this study, we have shown that such amino acid residues are Ala296_C and Ala296_B. Previously, we performed docking using the A740003 antagonist as a template in the allosteric site. In this work, the A438079 blocker was docked to the allosteric site, and then the A438079 was used as a template for docking **U-556**. This resulted in **U-556** making contact with Ala296_B, as did A438079, and additionally with Ala296_C.

In our study, we found the partial inhibition of P2X7R by **U-556** and A438079. The incomplete blocking of the receptor by A438079 has been described in a number of papers. For instance, in adenocarcinoma cells, this selective blocker had an unimpressive effect on ATP-induced P2X7R pore formation and cell death [21]. Partial blocking of receptor function by the investigated 1,4-naphthoquinone **U-556** may be of a similar nature to the A438079 blocker since both of these compounds bind to the same allosteric site of the receptor.

The process of carrageenan-induced paws edema develops over time and is a biphasic process. In the early phase, histamine, serotonin, and bradykinin are the first mediators involved, whereas prostaglandins, COX-2, various cytokines, such as IL-1β, IL-6, IL-10, and TNF-α, reactive oxygen species, and hydroxyl radicals are implicated in the second phase [22]. Notably, the maturation and implementation of the pro-inflammatory cytokines depend on the canonical activation of the NLRP3 inflammasome through Pannexin-1/P2X7/K^+^ efflux signaling. Blocking P2X7R by specific ligands or using macrophages of knockout *P2rx7^−/−^* mice resulted in a significant decrease in the carrageenan effect [23]. In our experiments, acute inflammation induced by carrageenan showed significant mitigation by **U-556** in the second phase after 4 and especially 24 h. Apparently, this is a consequence of the conjugate **U-556** impact on P2X7R followed by inhibition of pro-inflammatory cytokine release and ROS generation responsible for paws edema. COX-2 inhibition by the studied compound described for some 1,4-NQs in our previous investigation [13] may also contribute to diminishing the edema rate in the second phase.

## 4. Materials and Methods

### 4.1. Synthesis of Tetracyclic Conjugate of 1,4-Naphthoquinone Thioglucoside (U-556)

Synthesis of 3-(tetra-O-acetyl-β-D-glucopyranosyl-1-thio)-5,8-dihydroxy-2-methoxy-6,7-dimethylnaphthalene-1,4-dione **3** was carried out by condensation of 2-chloro-5,8-dihydroxy-3-methoxy-6,7-dimethylnaphtalene-1,4-dione **1** with tetra-O-acetyl-1-thio-β-D-glucose **2** according [12]. Acetylated thioglucoside **3** was characterized as red solid with R*_f_* 0.43 (hexane-benzene-acetone, 2:1:1 v/v, silufol plates), yield 81%, mp 169–171 °C. ^1^ H NMR (700 MHz, CDCl_3_): δ 1.95 (s, 3H), 2.01 (s, 3H), 2.02 (s, 3H), 2.08 (s, 3H), 2.26 (s, 3H), 2.27 (s, 3H), 3.70 (ddd, 1H, *J* 2.4, 5.1, 10.0 Hz), 4.06 (dd, 1H, *J* 2.4, 12.3 Hz), 4.15 (m, 1H, *J* 5.1, 12.3 Hz), 4.21 (s, 3H), 5.09 (dd, 1H, *J* 9.3, 10.0 Hz), 5.11 (dd, 1H, *J* 9.3 10.0 Hz), 5.26 (dd, 1H, *J* 9.3, 9.4 Hz), 5.50 (d, 1H, *J* 10.0 Hz), 12.06 (s, 1H), 13.41 (s, 1H). ^13^C NMR (CDCl_3_, 176 MHz): δ 12.33, 12.57, 20.48, 20.55, 20.58, 20.67, 61.91, 62.06, 68.30, 71.23, 73.99, 75.94, 82.06, 107.95, 109.70, 126.98, 139.62, 140.81, 159.52, 165.29, 166.25, 169.34, 169.41, 170.17, 170.49, 173.24, 178.13.

Tetracyclic conjugate **4** (**U-556**) was prepared according to [12] by treatment **3** with MeONa solution in dry MeOH. The red precipitate of **4** was filtered off, dried, and yielded (2R,3S,4S,4aR,12aS)-3,4,7,10-tetrahydroxy- 2-hydroxymethyl-3,4,4a,12a-tetrahydro-2H-naphtho[2,3-b]pyrano[2,3-e][1,4]oxathiine-6,11-dione (**4**); R*_f_* 0.47 (benzene-ethylacetate- methanol, 7:4:2 v/v, silufol plates), yield 80%, mp 314–316 °C [21]. ^1^H NMR (500 MHz, DMSO-_d6_): δ 2.18 (s, 3H), 2.19 (s, 3H), 3.32 (m, 1H), 3.50 (m, 2H), 3.60 (m, 2H), 3.75 (m, 1H), 4.81 (m, 1H), 5.01 (m, 1H), 5.49 (d, 1H, *J* 5.9 Hz), 5.76 (m, 1H), 12.64 (s, 1H), 12.94. (s, 1H). ^13^C NMR (DMSO-_d6_, 125 MHz): δ 12.1, 12.2, 60.7, 70.4, 73.4, 73.9, 79.3, 82.3, 117.3, 107.4, 123.5, 137.8, 137.9, 150.4, 156.3, 157.3, 177.7, 183.0 [14].

The total scheme of compound **U-556** synthesis is shown in Figure 1.

Compound **U-556** was dissolved in 100% DMSO at a concentration of 10 mM and stored as a stock solution. Intermediate stock solutions were then prepared at a concentration of 100 μM in 10% DMSO. For experiments, **U-556** solution was added in a volume of 20 µL per well of 96-well plates to 180 µL of cell medium so that the final concentration of DMSO in the incubation medium did not exceed 1%.

### 4.2. Cell Line

Mouse macrophage cell line RAW 264.7 TIB-71™ was purchased from ATCC (American Type Culture Collection, Manassas, VA, USA). Cells were grown in DMEM medium (Biolot, St. Petersburg, Russia) supplemented with 10% fetal bovine serum (Biolot, St. Petersburg, Russia) and 1% penicillin/streptomycin (Biolot, St. Petersburg, Russia) in a CO_2_ incubator at 37 °C and 5% CO_2_.

### 4.3. Ca^2+^ Influx Measurement

RAW 264.7 cells were seeded in 96-well plates (4 × 10^4^ cells per well) in a DMEM cultural medium and incubated overnight at 37 °C, 5% CO_2_. Then, the cells were washed once with HBSS saline (140 mM NaCl, 5 mM KCl, 0.8 mM MgCl_2_, 2 mM CaCl_2_, 10 mM glucose, 10 mM HEPES, pH 7.4) and loaded with 5 μM Fluo-8 AM (Abcam, Cambridge, UK) and 0.05% (w/v) Pluronic F-127 (Sigma-Aldrich, Burlington, MA, USA) in the same buffer solution. Cells were incubated for 40 min (37 °C, 5%) and then were washed with HBSS buffer without the fluorescent dye and treated by studied compounds for 20 min at RT in the dark. ATP (Sigma, Burlington, MA, USA) was used as an agonist of P2X7R. Competitive P2X7R antagonist A438079 (10 μM, Sigma, Burlington, MA, USA) were used as inhibitory controls. Ionomycin (Sigma-Aldrich, St. Louis, MO, USA) was used to generate a generic calcium signal in cells. Fluo-8 was excited at 485 nm, and the emission at 520 nm was measured with a PHERAstar FS plate reader (BMG LABTECH, Ortenberg, Germany). The data were processed by MARS Data Analysis v. 3.01R2 (BMG Labtech, Ortenberg, Germany). Mean values and standard error of peak height calcium were calculated.

### 4.4. YO-PRO-1 Uptake Assay

RAW 264.7 cells were seeded in 96-well plates at a density of 4 × 10^4^ cells/well and incubated in DMEM for 24 h, 37 °C, 5% CO_2_. The cells were then washed twice with HBSS, and the medium was changed to the same buffer containing YO-PRO-1 dye (Sigma, Burlington, MA, USA, 2.5 μM final concentration). Further, the studied compound was added to the cells at concentrations of 5, 1, and 0.1 μM. P2X7 receptor blocker A438079 at a concentration of 10 μM was used as a comparison control. Two mM ATP (Sigma, Burlington, MA, USA) or 200 μM 2,3-O-(4-benzoylbenzoyl)ATP (BzATP, Sigma-Aldrich, St. Louis, MO, USA) were used as an agonist of P2X7R. In some experiments, cells were treated with 350 μM oxidized ATP (OxATP, Sigma-Aldrich, St. Louis, MO, USA) for 2 h. The YO-PRO-1 fluorescence was measured continuously every 100 sec for 800 sec after adding ATP or BzATP with a PHERAstar FS plate reader (BMG Labtech, Ortenberg, Germany) at λex = 480 nm and λem = 520 nm, ATP 2 mM or BzATP 200 μM were added after baseline recording and HBSS (20 μL) was added as a negative control.

### 4.5. ROS Formation Assay

RAW 264.7 cells were seeded in 96-well plates at a density of 4 × 10^4^ cells per well for 24 h. The medium was replaced with fresh DMEM and treated with LPS (100 ng/mL) for 3 h at 37 °C. After that, the cells were treated with the studied compound (5.0, 1.0, 0.1 μM final concentrations) for an additional 30 min. P2X7R blocker A438079 (10 μM) was used as inhibitory control. Then ATP (300 μM final concentration) was added to each well for 30 min. For fluorescence measurement, the cells were washed once, and the cell medium was replaced by HBSS, containing 10 μM of fluorescent dye 2,7-dichlorodihydrofluorescein diacetate (H_2_DCFDA) (Sigma, Darmstadt, Germany). Cells were incubated for 30 min at 37 °C, washed twice with HBSS without fluorescent probe, and additionally incubated for 20 min at RT in the dark. The measurement of fluorescence intensity was carried out using PHERAstar FS plate reader (BMG Labtech, Ortenberg, Germany) at λex = 485 nm and λem = 520 nm.

### 4.6. Cytoprotection Activity Assay

#### 4.6.1. Detection of Lactate Dehydrogenase (LDH) Release

RAW 264.7 cells (4 × 10^4^ cells/well of a 96-well plate) were seeded and incubated for 24 h at 37 °C, 5% CO_2_ in an incubator. Then, the cells were treated with **U-556** at final concentrations of 5.0, 1.0, and 0.1 μM and incubated for 1 h. A438079 (10 μM) and apyrase (30 U/mL) were used as inhibition control. Next, LPS (100 ng/mL, Sigma, St. Louis, MO, USA) was added to the cells, and the cells were incubated for 4 h. Thereafter, ATP (2 mM final concentration) was added to the cells, and the plates were incubated for an additional 1 or 24 h. Cells incubated without ATP and LPS were used as negative controls. The plate was then centrifuged at 250× *g*, and 100 μL of the supernatant from each well was transferred to the relevant wells of an optically clean 96-well plate. An equal volume of the reaction mixture (100 μL) from the LDH Cytotoxicity Assay Kit (Abcam, Cambridge, UK) was added to each well and incubated for 30 min at room temperature. The absorbance was measured at λ = 490 nm using a Multiscan FC spectrophotometer (Thermo Scientific, Nummela, Finland).

#### 4.6.2. FDA Cell Viability Assay

A stock solution of fluorescein diacetate (FDA) (Sigma-Aldrich, St. Louis, MO, USA) in DMSO (1 mg/mL) was prepared. RAW 264.7 cells were seeded and treated as described above. Further, FDA solution (50 μg/mL) was added to each well, and the plate was incubated in the dark at 37 °C for 15 min. Cells were washed, and fluorescence intensity corresponding to nonspecific esterase activity was measured using a Fluoroscan plate reader (Thermo Labsystems, Helsinki, Finland) at λex = 485 nm and λem = 518 nm. Cell viability was expressed as a percentage of control.

#### 4.6.3. MTT Assay

RAW 264.7 cells (4 × 10^4^ cells/well) were seeded and incubated in 96-well plates for 24 h at 37 °C and 5% CO_2_ in an incubator. Compound **U-556** was added to the wells at different concentrations, and the plates were incubated for an additional 1 h. A438079 (10 μM) and apyrase (30 U/mL, Sigma, Burlington, MA, USA) were also added to cells as standard blockers of ATP action. Then ATP (2 mM, final concentration) and LPS (100 ng/mL, final concentration) were added to the cells. Cells were incubated for 24 h, after which cell viability was determined using the MTT reagent (3-(4,5-dimethylthiazol-2-yl)-2,5-diphenyltetrazolium bromide, Sigma-Aldrich, Saint-Louis, MO, USA). For this purpose, 10 μL of MTT stock solution (5 mg/mL) was added to each well, and the microplate was incubated for 4 h at 37 °C. After that, 100 μL of SDS-HCl (1 g SDS/10 mL dH2O/17 μL 6 N HCl) were added to each well, followed by incubation for 4–18 h. The absorbance of the converted dye formazan was measured using Multiskan FC microplate photometer (Thermo Scientific, Waltham, MA, USA) at a wavelength of 570 nm. The results were presented as percentage of control data. The tested compound was added to the wells of the plates in a volume of 20 μL dissolved in PBS (DMSO concentration > 1%).

#### 4.6.4. Trypan Blue Exclusion Cell Viability Test

RAW 264.7 cells (4 × 10^4^ cells/well of a 96-well plate) were seeded and incubated for 24 h at 37 °C, 5% CO_2_ in an incubator. Then, the cells were treated with LPS (100 ng/mL) and inhibitors, as described above. After 24 h of incubation with ATP (2 mM), the cells were resuspended in the medium of each well. Ten µL of the cell medium containing the cell suspension was mixed with 10 µL of 0.4% trypan blue. The mixture was loaded onto counting slides, and cell viability was assessed using the TC20 automated cell counter (Bio-Rad, Hercules, CA, USA).

### 4.7. Radical Scavenging Assay

DPPH radical scavenging activity of compounds was tested as described [24] with minor modifications. The compounds were dissolved in MeOH, and the solutions of conjugate **U-556**, ascorbic acid (Vekton, St. Petersburg, Russia), or quercetin (Sigma-Aldrich, Steinheim, Germany) as a positive control (120 µL) were dispensed into wells of a 96-well microplate. In all of them, 30 µL of the DPPH (Sigma-Aldrich, Steinheim, Germany) solution in MeOH (0.75 mM) was added to each well. The concentrations of compounds and ascorbic acid in the mixture were 0.01–100.0 µM. The plates were incubated in the dark at room temperature for 30 min, and then the absorbance was measured at 517 nm with a Multiskan FC microplate photometer (Thermo Scientific, Waltham, MA, USA). The negative control contained no test compound. The final results were reported as DPPH radical scavenging (%) in the reaction solution.

### 4.8. Determination of Antioxidant Activity in Brain Homogenate

The procedure was performed according to the protocol described in [25]. The brain of female C57BL/6 mouse was washed with cold buffer (140 mM KCl, 10 mM K_2_HPO_4_, pH 7.4) and then homogenized using a Potter–Elehjem tissue homogenizer, adding the buffer to the brain in a weight ratio of 1:4. 10 mL of buffer was added to the resulting homogenate and centrifuged for 10 min at 900 rpm in a Labofuge 44R centrifuge (Thermo Scientific, Karlsruhe, Germany). The supernatant was transferred to a volumetric flask and adjusted to 25 mL with a buffer. The Lowry protein concentration in homogenate was in the range of 0.27–0.3 mg/mL. To determine the antioxidant activity, the solution of the tested compound was added to the brain homogenate, then 2 mM FeSO_4_ solution was added, and the reaction was stopped by adding of 20% trichloroacetic acid (TCA) (Reachim, Moscow, Russia). The homogenate was centrifuged, supernatant was collected, and 0.7% solution of thiobarbituric acid (TBA) (Sigma, Saint-Louis, MO, USA) in 50% glacial acetic acid was added to reveal the products reacting with TBA. The content of TBA-reacting substances (TBARS) was determined spectrophotometrically using PHERAstar FS plate reader (BMG Labtech, Ortenberg, Germany) by measurement of fluorescence intensity at 480/520 nm (λex/λem).

### 4.9. Surface Plasmon Resonance

SPR analysis was performed at 25 °C using a Biacore^®^ 3000 optical biosensor and CM5 sensor chips (GE Healthcare, Chicago, IL USA). Buffer solution HBS-N (10 mM HEPES, 150 mM NaCl, pH 7.4) (Cytiva, Chicago, IL, USA) was used as a working buffer. Protein hP2X7 (47–334 a.a., extracellular domain, 36.8 kDa) (abx167020 Abbexa LTD, Cambridge, UK) was covalently immobilized on the surface of the chip by forming amide bonds between dextran carboxyl groups and free amino groups of the protein. The carboxyl groups of the chip were activated for 5 min by injecting a mixture 1:1 *v/v* of 0.2 M 1-ethyl-3-(3-dimethylaminopropyl)carbodiimide hydrochloride (EDC) and 0.05 M N-hydroxysuccinimide (NHS) at a rate of 5 μL/min followed by a 1 min wash with HBS-N buffer at the same flow rate. Next, hP2X7 (15 μg/mL) in 10 mM sodium acetate (pH 5.0) was injected into the working channel of the biosensor for 10 min at a flow rate of 5 μL/min. The final level of immobilization was 12,300 RU (12.3 ng of protein). The control channel without immobilized P2X7R was used to correct the effects of the nonspecific binding of analytes to the chip surface. Compound **U-556** was prepared as 1 mM stock solutions in DMSO and then was dissolved in HBS-N buffer (1:100 *v*/*v*). The samples were injected through the channels of the biosensor (working and reference) at a flow rate of 10 μL/min for 6 min. The dissociation of the formed 1,4-NQ/P2X7 complexes was recorded for at least 6 min at the same flow rate after the injection of the sample. After each cycle of measurement, the bound analyte was removed by double injection of the regenerating solution (2 M NaCl, 1% CHAPS) at a flow rate of 30 μL/min for 30 s.

SPR sensorgrams were processed in Biacore 3000 Evaluation Software v.1.0 (GE Healthcare, Chicago, IL, USA) and BIAevaluation Software v. 4.1.1 (GE Healthcare, Chicago, IL, USA) using “1:1 binding (Langmuir)” model. The 1:1 (Langmuir) binding model is a 1:1 interaction model between a compound (C) and an immobilized protein (P) and is equivalent to the Langmuir isotherm for surface adsorption: C + P ↔ CP. The final kinetic parameters were obtained from the fits that best agree with the experimental curves for the minimum of the obtained value of chi2. Chi2 is a measure of the average squared residual between the experimental data and the fitted curve. The equation describing the interaction model used is as follows: (1) 1:1 (Langmuir) binding:

Kd = koff/kon,

where Kd is the equilibrium dissociation constant, koff is the dissociation rate constant, kon is the association rate constant.

### 4.10. Molecular Modeling

The homology model of the mouse P2X7R trimer (UniProt ID Q9Z1M0) in the closed form was built with the Homology module of the Molecular Operating Environment (MOE) program [26], using as a template structure of the rat P2X7R closed form (PDB ID 6U9V). Energy minimization of the model mP2X7R trimer with force field Amber10:EHT and 3D-protonation were carried out using MOE 2020 program. The 3D structure of **U-556** was generated with the Build module of MOE program using MMFF94 potential for structure optimization. The molecular docking of compounds to the model of mP2X7R trimer was carried out using SiteFinder and Dock modules of the MOE program. Template docking of A438079 was carried out using P2X7R antagonist A740003 binding site as template and refinement 5 poses with Induced Fit and Score GBVI/WSA dG. Template docking of **U-556** was carried out using P2X7R antagonist A438079 binding site as a template and Score GBVI/WSA dG. Analysis of ligands contacts was carried out using Ligand Interaction module of the MOE program.

### 4.11. Carrageenan-Induced Paw Edema

Mouse paw edema, an acute inflammatory model, was used to investigate the antagonistic action of **U-556**. The female C57BL/6 mice were divided into groups of 6 individuals, each mouse was measured by the control volume of the paw using a plethysmometer (Ugo basile 37140, Milan, Italy). Each group then received tested 1,4-naphthoquinone diluted in sterile H_2_O from a stock solution in DMSO (10 mM) at different dosages. The final concentration of DMSO was 1%. In these experiments, 1,4-naphthoquinone at dosages of 0.1, 1.0, and 10.0 mg/kg was injected intraperitoneally at 60 min prior to carrageenan-σ (Sigma, Saint-Louis, MO, USA) administration. Control mice received an intraperitoneal injection of the solvent. Carrageenan in saline solution at a concentration of 1.5 mg/mL in the volume of 20 μL was injected into the back paw. After 1, 2, 4, 6, and 24 h, paw edema was measured using a plethysmometer. The volume of the paw before the start of the experiment was taken as 100%.

### 4.12. Animals

Female C57BL/6 mice (two months of age, 20–22 g) purchased from the Russian National Center for Genetic Resources of Laboratory Animals on the basis of the SPF vivarium (ICiG SB RAS, Novosibirsk, Russia) were used for experiments. All mice were raised in an environment with a 12-h light/dark cycle at a temperature of 22 ± 1 °C with available food and water. All experiments were conducted according to the International Recommendations for Biomedical Research Using Animals, adopted by the International Council of Medical Scientific Societies (CIOMS) and the Order of the Ministry of Health and Social Development of Russia dated 23.08.2010 No. 708n “On Approval of Laboratory Practice Rules”.

### 4.13. Statistics

All data were obtained in independent replicates, and calculated values were expressed as mean ± standard error of the mean (SEM). The Student’s *t*-test was performed using SigmaPlot 14.0 (Systat Software Inc., San Jose, CA, USA) to determine statistical significance.

## 5. Conclusions

In summary, purinergic P2X7 receptors, which now are associated with a series of important functions in normal and pathophysiology, are known as molecular targets, useful in the search for new low-molecular-weight pharmaceutical leads. Herein, we have convincingly shown that the studied small molecule is able to bind P2X7R and modulate its functions, which ultimately leads to cell protection from the harmful ATP action and the anti-inflammatory effect. Obviously, all our findings of **U-556** activity strengthen its therapeutic potential and the feasibility of developing new P2X7R inhibitors based on the 1,4-naphthoquinone pharmacophore group.

## Figures and Tables

**Table 1:** DPPH free radical scavenging by quinone-thioglucoside conjugate **U-556**.

Compound	DPPH Radical Scavenging, %				
**U-556**	**0.1 μM**	**1.0 μM**	**5.0 μM**	**50.0 μM**	**100 μM**
	7.74 ± 2.01	9.46 ± 3.51	21.43 ± 3.79	34.69 ± 0.46	48.03 ± 1.36
**Quercetin**	6.49 ± 0.49	13.07 ± 0.87	27.30 ± 0.90	45.27 ± 0.61	61.24 ± 0.28
**Ascorbic acid**	14.20 ± 0.34	16.01 ± 2.27	35.21 ± 0.30	82.08 ± 0.71	97.09 ± 1.49

**Table 2:** Kinetic and equilibrium parameters of the U-556/hP2X7R complex.

Compound	kon, mole-1 c-1	koff, C-1	Kd, M	Evaluation Model
**U-556**	(1.47 ± 0.18) × 10^−2^	(1.91 ± 0.21) × 10^−3^	13.0 × 10^−6^	Langmuir 1:1

**Table 3:** Amino acid contacts of quinone-thioglucoside conjugate **U-556** and A438079 with the mP2X7R homotrimer extracellular domain.

Compound		Ligand		Receptor			Interaction	Distance	E (kcal/mol)
**U-556**		04	23	O	ALA 296	(C)	H-donor	2.63	−1.2
		06	17	N	ALA 296	(C)	H-acceptor	3.42	−0.6
		04	23	OH	TYR 298	(A)	H-acceptor	3.13	−0.8
		0	45	N	ALA 296	(B)	H-acceptor	2.98	−3.0
**A438079**	N7		7	N	ALA 296	(B)	H-acceptor	3.27	−2.6
	6-ring			6-ring		(A)	pi-pi	3.55	−0.0

## Data Availability

The data presented in this study are available upon request from the corresponding author.

## Abbreviations

1,4-NQ	1,4-Naphthoquinone
ATP	Adenosine-5’-triphosphate
BzATP	2,3-O-(4-benzoylbenzoyl)ATP
OxATP	oxidized ATP
DMEM	Dulbecco’s Modified Eagle Medium
DMSO	Dimethylsulfoxide
DPPH	2,2-Diphenyl-1-picrylhydrazyl
FDA	Fluorescein diacetate
H2DCF-DA	2,7-Dichlorodihydrofluorescein diacetate
HEPES	(4-(2-hydroxyethyl)-1-piperazineethanesulfonic acid
LDH	Lactate dehydrogenase
LPS	Lipopolysaccharide
NHS	N-hydroxysuccinimide
TBARS	thiobarbituric acid reacting substanses
PBS	Phosphate-buffered saline
P2X7R	Purinergic receptor of P2X family
ROS	Reactive oxygen species
SDS	Sodium dodecyl sulfate
SPR	Surface plasmon resonance
